# Impact of adhesive capsulitis on quality of life in elderly subjects with diabetes: A cross sectional study

**DOI:** 10.4103/0973-3930.45272

**Published:** 2008

**Authors:** Saumen Gupta, Kavitha Raja, N Manikandan

**Affiliations:** Department of Physical Therapy, Manipal College of Allied Health Sciences, Manipal University, Manipal, India

**Keywords:** Adhesive capsulitis, diabetes, elderly, quality of life, SF-36

## Abstract

**CONTEXT:**

Adhesive capsulitis (AC) is a common musculoskeletal manifestation in elderly having long standing history of diabetes. This hinders the function of shoulder which is crucial in many activities of daily living. This painful, functional deficit may decrease the quality of life in elderly.

**AIMS:**

The purpose of this study was to evaluate the impact of AC on quality of life in diabetic elderly subjects.

**SETTINGS AND DESIGN:**

District government Hospital, Udupi; Dr. T. M. A. Pai, Udupi, Kasturba Hospital, Manipal; and the study design is cross-sectional design.

**MATERIALS AND METHODS:**

Two hundred and thirty-three diabetic elderly patients were recruited from the settings based on cluster sampling. They were evaluated for pain and restriction of range of motion in the shoulder joint and were. Severity of condition was classified on Oxford shoulder score and quality of life was calculated by SF- 36. Statistical analysis used: Descriptive statistics and spearman's correlation coefficient done in SPSS.

**RESULTS:**

Forty-nine percent of women had diabetic AC. Majority of the subjects without AC fell in average quality of life. Women with AC fell in unhealthy category, whereas men were clustered in the average category.

**CONCLUSIONS:**

Adhesive capsulitis was an important factor in reducing the quality of life of the elderly with diabetes.

## Introduction

Decreased shoulder mobility has serious functional implications in the elderly as the shoulder is a very complex joint that is crucial to many activities of daily living. Adhesive capsulitis (AC) or frozen shoulder (FS) is a condition characterized by an insidious and progressive loss of active and passive mobility of glenohumeral joint, presumably due to capsular contracture.[1] The estimated prevalence of AC is 11%–30% in diabetic patients, which is considerably greater than that in non-diabetics.[2] AC has been associated with the duration of diabetes and age with diabetics experiencing significantly greater pain and dysfunction.

Pain and function in AC can be reliably assessed with the Oxford Shoulder Score and quality of life can be assessed reliably by SF-36.[3–5] These evaluation tools could be applied to patients with AC and may help to identify patients who potentially require a longer treatment course or those whose outcome will be less satisfactory.

### Oxford Shoulder Score

Oxford shoulder score (OSS) is a validated patient-completed outcome measure specifically designed for the evaluation of patients suffering from shoulder pathology other than instability. Studies report that patients have found this questionnaire to be easy to complete, and it provides reliable, valid and responsive data regarding their perception of shoulder problems. The construct validity of the questionnaire has been previously reported. The items are internally consistent and reproducible and the questionnaire may therefore be considered to be reliable as a clinical measure of outcomes.[3–5]

### Short Form-36

Short form-36 (SF-36), a generic quality of life questionnaire, measures patients' general health status. The SF-36 contains multi-function item scales to measure eight domains: physical function (10 items); role physical (four items); bodily pain (two items); general health (five items); vitality (four items); social functioning (two items); role emotional (four items); and mental health (five items). The two summary measures of the SF-36 are the physical component summary and the mental component summary. The SF-36 can be self-administered or interview-administered and takes less than 15 minutes to complete. The reliability of the eight scales and two summary measures has been estimated using both internal consistency and test-retest methods and is high (*r* = 0.8).

Based on general population norms, all scores above or below 50 ± 10 can be interpreted as above or below the general population norm, i.e., a score of 1.96 standard deviations above or below the mean would suggest that, with 95% confidence, the sample is healthier or unhealthier than are people in general on that measure.[6,7]

Compared with the eight SF-36 scales, scores for physical and mental health summary measures can be estimated with smaller confidence intervals, expand the range of health states measured, and greatly increase the number of levels distinguished in comparison with any one of the eight scales. While the summary measures do not reproduce all of the reliable variance in the eight-scale SF-36 profile, they have the advantage of reducing the number of statistical comparisons required when analyzing SF-36 data. Empirical tests suggest that they do so without a substantial loss of information. This pattern of correlations between scales and summary component scores is also quite robust, suggesting that each summary has a comparable interpretation across population subgroups.

The objective of this study was to study the impact of AC on quality of life in diabetic elderly.

## Materials and Methods

Two hundred and thirty-three patients from hospitals in Udupi were evaluated for diabetes and AC. The inclusion criteria for subjects were as follows: symptomatic shoulder problems in elderly with diabetes mellitus (age ≥ 60 years). The exclusion criteria were (1) Any neurological conditions affecting shoulder. (2) Any musculoskeletal pathology in upper limb other than AC. (3) Surgery of head, neck or upper limb.

### Procedure

The study was reviewed and approved by the institutional research committee of the Manipal College of Allied Health Sciences, Manipal University, Manipal. Prior to subject selection permission for recruitment of subjects from outdoor patient department (OPD) of the department of medicine was sought from medical superintendent of the selected hospitals. Elderly patients with diabetes who came to medical OPDs during the study period were interviewed regarding their shoulder function after obtaining informed consent.

After the subject's diabetic history was confirmed from the medical chart, subjects were assessed for pain and restriction of range of motion in their shoulder. Diabetic elderly subjects who had pain and/or restriction of range of motion were referred to the orthopaedic surgeon for diagnosis. Those subjects, who were diagnosed as AC, were classified according to severity based on responses of OSS into mild, moderate and severe. Patient's pain was recorded using the visual analogue scale (VAS). Dominance of the hand was noted. All the subjects were given SF-36 survey questionnaire to record their quality of life. Those subjects who were unable to read or write were interviewed and their responses were marked by the tester to report their quality of life. Scoring for SF-36 subscale component was done by online software.[8]

### Outcome measures

Measures for reporting used were as follows:

1. Demographics – prevalence of AC
2. Severity of AC – Oxford Shoulder Score
3. Quality of life – SF-36
4. Pain severity – VAS

### Data analysis

Statistical analysis of the data was done by using the SPSS statistical package version 11.5. Descriptive statistics was used to calculate the prevalence of AC.

Spearman's correlation coefficient was used to determine correlation of:

- quality of life with age;
- severity of AC with quality of life; and
- severity of pain and quality of life.[7]

## Results

The demographic characteristics of subjects who participated in the study are described in Table 1. Subjects were classified into diabetics with and without AC in order to compare their QOL.

**Table 1:** Demographic characteristics of subjects with diabetes mellitus, with and without AC

Diagnosis	Gender	Age (year) mean ± SD	Duration of DM (year) mean ± SD
With AC	Male(*n* = 24)	66.33 ± 5.81	11.5 ± 9.18
Female	(*n* = 45)	66.84 ± 6.18	7.67 ± 5.56
Without AC	Male(*n =* 102)	68.05 ± 7.07	9.51 ± 8.38
Female	(*n* = 62)	65.98 ± 6.08	8.00 ± 5.95

Table 1 shows that the mean age of the two groups were comparable. There was slight difference in duration of diabetes in male individuals.

42% of women and 19% of men of the total population of men and women with diabetes had AC. 79.71% of the subjects had AC on the dominant side (80% were men and 77.77% were women).

In the following table, the subjects with AC were categorized based on OXFORD SHOULDER SCORE into mild (21-30), moderate (31–40), severe groups (41–60). Twenty-six subjects had a normal score on the OSS but complained of painful restriction in the capsular pattern in shoulder (median VAS score– 4). These individuals were classified as early stage of adhesive capsulitis.” The mean age of these individuals was as follows: men – 65.84 ± 5.74 years, women – 68.53 ± 6.43 years and duration of diabetes in men – 10.69 ± 7.52 years and women – 7.75 ± 6.51 years.

Table 2 describes the characteristics of the three groups. There was no discernable relationship between severity of AC and age. Men with AC had longer duration of diabetes than women. In the severe group, there was no mean difference between genders. There was a direct relationship between severity (OSS) and median VAS, especially evident in women.

**Table 2:** Demographic data of subjects with AC categorized according to Oxford Shoulder Score

Oxford shoulder score category	Gender	Age (year) mean ± SD	Duration of DM (year) mean ± SD	Median VAS*
21–30 (mild)	Male(*n* = 3)	66.33 ± 5.68	16.33 ± 11.84	4
	Female(*n =* 12)	66.33 ± 5.61	6.6 ± 5.54	5
31–40 (moderate)	Male(*n* = 4)	68.5 ± 9.32	12.75 ± 10.01	5.5
	Female(*n =* 11)	64.36 ± 5.51	5.88 ± 2.93	5
41–60 (severe)	Male(*n* = 4)	65.75 ± 3.30	9.25 ± 13.86	6.5
	Female(*n =* 9)	68.11 ± 7.16	10.55 ± 5.91	8

* VAS – visual analogue scale for recording of pain

SF-36 scores were computed for subjects based on shoulder status. Only physical component summary score and mental component summary score were considered for analysis.[9,10] These scores based on severity of AC are described below in Table 3.

**Table 3:** SF 36 score of subjects based on severity of AC

	Age (year) mean ± SD	Gender	Physical component summary	Mental component summary
Without AC	67.27 ± 6.77	Males(*n* = 102)	47.42 ± 8.30	50.91 ± 8.58
		Females(*n* = 62)	48.52 ± 8.35	51.67 ± 7.27
Mild	66.33 ± 5.42	Males(*n* = 3)	34.49 ± 11.49	38.56 ± 12.56
		Females(*n* = 12)	40.85 ± 8.24	47.54 ± 11.73
Moderate	65.46 ± 6.63	Males(*n* = 4)	35.05 ± 7.03	42.10 ± 12.99
		Females (n = 11)	38.38 ± 10.31	47.96 ± 8.96
Severe	66.38 ± 6.18	Males (n = 4)	28.77 ± 7.61	35.24 ± 5.91
		Female (n = 9)	30.34 ± 8.01	33.10 ± 10.20

As can be seen from Table 3, the physical component summary decreased for both men and women based on severity of AC. Men reported lower scores as compared to their female peers. However, mental component summary did not show a similar trend. But there was significantly lower score reported by the subjects with AC when compared with those who did not have AC (PCS – *P* = 0.004, MCS – *P* =0.000). There was no correlation between the age and the quality of life.

There was moderate negative correlation between severity based on OSS and quality of life among males on the physical component summary of SF-36; *r* = −0.73, while correlation between OSS and mental component summary of SF-36: was poor (*r* = 0.5). There was minimal correlation among women between severity based on the OSS and the quality of life physical component summary; *r* = 0.4, mental component summary; *r* = 0.5.

Norms for the SF-36 have been standardized for physical and mental component scores, and a mean of 50 ± 10 has been suggested to indicate average levels of health. It has been suggested that scores of greater than average may indicate greater health and less than average, poorer health. Based on this, we classified subjects into healthy, average and unhealthy. The SF-36 scores based on this categorization are depicted in Table 4.

**Table 4:** Distribution of subjects with (*n* = 69) and without AC (*n*= 164) in quality of life categories

		Physical component summary score		Mental component summary score	
			
		With AC	Without AC	With AC	Without AC
Healthy	M	0	0	1 44%	4 87%
	F	0	0	2.89%	3.04%
Average	M	18.84%	50.61%	23.18%	49.39%
	F	28 98%	32 92%	39 13%	32 31 %
Unhealthy	M	15.94%	11.58%	10.14%	7.92%
	F	36.23%	4.87%	23.18%	2.43%

As can be seen from the above table, the majority of the subjects with AC fell in the unhealthy category in the physical component summary scores. This was more evident in women, whereas men were clustered in the average category. This was not as evident in the mental component summary score. Majority of the subjects without AC fell in average category.

## Discussion

In this study, the overall prevalence of AC in diabetic subjects was noted to be 29.61%. As per literature, AC has a prevalence of 2% in the general population, but is reported to occur in 10% to 29% of those with diabetes. The results of this study show a similar prevalence.

Shoulder capsulitis is common in type 1 and type 2 diabetic patients. It is associated with age in type 1 and 2 diabetic patients and with the duration of diabetes in type 1 patients. Noninsulin dependent diabetics also have an increased incidence of frozen shoulder, but not as high as in insulin users. In this study, all subjects were type 2 diabetics and hence no comparison could be made. An increased prevalence of AC was noted in female diabetic subjects (42%). Several authors have confirmed increased prevalence of diabetic AC in females.

It was observed that the majority of the subjects had AC on the dominant side, (80% for men and 77.77% for women). Most of the studies have demonstrated higher incidence of AC in nondominant arm. The reasons for this difference, although unconfirmed may stem from reasons of activity, which is more often of greater intensity on the dominant side. This has been suggested by De Palma as well.[1]

When the duration of diabetes was looked into, men had longer duration of diabetes except in the severe AC category, where the genders were matched. Published studies have stated that AC of the shoulder is associated with duration of diabetes. We could not find any literature on gender differences in this factor. It is of note that most subjects in our study were from a rural background and diabetes was most often an incidental finding when the patient sought medical treatment for other acute illnesses. Hence, the duration of diabetes, reported by the subjects may not always have been accurate. All subjects with AC in this study had DM for > 5 years. These may also have contributed to the absence of age related findings.

The data from this study documents the notable impact of AC on quality of life. Subjects with AC reported a significantly lower score on both components summaries of SF-36 than those without AC. It was interesting to note that quality of life measurement indicated by SF-36 score showed that physical component summary decreased based on severity of AC. This trend was especially evident in women. However, mental component summary did not show a similar trend. Several studies of the validity and usefulness of outcomes instruments have confirmed the concept that physical impairment interacts with the social and emotional aspects of general health. Gartsman *et al*. demonstrated that five common conditions affecting the shoulder, including AC, had a significant impact on scores on the Short Form-36, a general health-status measure. Their study showed significant differences in the scores for physical function, physical role, comfort/pain, vitality, social function, and emotional role for patients with AC when compared with norms for the general population.

When comparison was made between the physical component summary and mental component summary of subjects with and without AC, it was seen that people without AC had higher quality of life scores in both the physical and mental components (*P* ≤ 0.000, *P* = 0.001). This certainly indicates that shoulder dysfunction directly impacts the person's quality of life. Although the dysfunction is of a physical nature, it appears to impact mental status as reported by the mental summary score. Variety of questions such as quantity of time the subject had problems with his work or other regular daily activities as a result of any emotional problems graded mental status in the physical functioning sphere. Hence, the physical dysfunction may have impacted the mental component score although not to a great extent.

The results may have been influenced by lack of cultural context in some aspects of SF-36. Culture can affect the perception and interpretation of health and illness and so may affect the responses to items in a questionnaire.[9]

When classified according to norm referenced categories, majority of the subjects without AC have rated their quality of life as average. In contrast, those subjects with AC presented a different picture. More women tended to be unhealthy, whereas men were clustered in the average category. But AC did not appear to have as much of an impact on the mental status of subjects as evidenced from the MCS scores. From this we can infer that while diabetes affected the subjects' quality of life, AC further increased the impact on the PCS.

### Implications and clinical significance of the study

Monitoring for signs of musculoskeletal complications can be an invaluable part of overall diabetes care. The results of this study implicate that shoulder dysfunction associated with diabetes has impact on the quality of life of elderly individuals with diabetes. Diabetic education programs must include shoulder screening, prevention and rehabilitation strategies for shoulder dysfunction. Literature has suggested that early intervention with mobility exercises may decrease the morbidity with AC. Clinicians should be aware of this possible musculoskeletal complication of diabetes in order to intervene early so that morbidity can be decreased. It has been demonstrated in literature that early intervention can decrease loss of mobility in AC.[11] As India has the dubious distinction of being the diabetic capital of the world, this aspect is doubly important for physiotherapists in India who work in public health systems.

### Limitations of study

As this was a cross-sectional study, number of episodes or the first onset of AC was not studied.

### Recommendations for further study

Diabetics should be followed in cohort to ascertain onset of shoulder dysfunction and associated risk factors. Prevalence based on age, severity and type of diabetes should be determined and the effectiveness of prevention strategies must be confirmed. Comparison of quality of life measures based on the above would also be interesting to ascertain.
